# Comparison of the Effectiveness of Paracetamol and Ibuprofen in the Management of Patent Ductus Arteriosus in Preterm Neonates: A Randomized Controlled Trial

**DOI:** 10.1186/s40348-025-00189-x

**Published:** 2025-01-25

**Authors:** S. Mohsin Ali Shah, Shaista Azeem Khan, Faran Sadiq, Ruba Gul, Faizan Sadiq, Misbah Ullah Khan, Muhammad Khalid Khan, Faryal Uzma, Arooj Khan, Sabir Khan

**Affiliations:** 1 Pediatrics Department, Khyber Teaching Hospital, Peshawar, Pakistan; 2 Accident and Emergency Department, Lady Reading Hospital, Peshawar, Pakistan; 3 Anesthesia Department, Lady Reading Hospital, Peshawar, Pakistan

**Keywords:** Patent ductus arteriosus, Paracetamol, Ibuprofen, Efficacy

## Abstract

**Background:**

Patent ductus arteriosus is one of the most common cardiac conditions affecting the neonates. Considering the lack of studies done on this topic in healthcare settings in Khyber Pakhtunkhwa province, this study aims to find out the comparative effectiveness of paracetamol and ibuprofen in management of PDA in our healthcare setting to conclude a better management option for the condition.

**Objective:**

To find and compare the effectiveness of paracetamol and ibuprofen in the closure of patent ductus arteriosus in preterm neonates.

**Methodology:**

This randomized controlled trial was conducted in the Department of Nursery and Neonatal Intensive Care Unit, Khyber Teaching Hospital, Peshawar, Pakistan, from 10th April 2024 to 10th October 2024. A total of 256 neonates of both genders with patent ductus arteriosus were included. Group A received oral paracetamol, and Group B received oral ibuprofen. The effectiveness of the treatments was evaluated at the end of the treatment period.

**Results:**

The age range in this study was from 48 to 96 h, with a mean age of 71.79 ± 13.10 h in Group A and 73.40 ± 11.81 h in Group B. Efficacy was observed in 107 (83.6%) patients in Group A compared to 90 (70.3%) patients in Group B, showing a statistically significant difference (*P* = 0.011).

**Conclusion:**

Our study has concluded that paracetamol is more effective than ibuprofen in closing patent ductus arteriosus. The trials were retrospectively registered at NIH Trial Registry (NCT06601114) https://clinicaltrials.gov/study/NCT06601114 dated 15/09/2024.

## Key points

**•** This study was conducted to find and compare the effectiveness of paracetamol and ibuprofen in the closure of patent ductus arteriosus in preterm neonates within the healthcare setting of Khyber Pakhtunkhwa Province, Pakistan.

**•** This study conclude that paracetamol is more effective than ibuprofen in closing patent ductus arteriosus (PDA) in preterm neonates.

**•** The higher efficacy rate observed with paracetamol suggests that it may be a preferable first-line treatment for PDA in this population.

## Introduction

Patent ductus arteriosus (PDA) is one of the most common cardiac conditions affecting the neonates. The ductus arteriosus (DA) provides a route to the predominant cardiac output in fetal life as the blood from heart is shunted from right to left via ductus arteriosus to systemic circulation. After birth, due to ventilation, the systemic vascular resistance increases & pulmonary vascular resistance decreases which increases pulmonary blood flow leading to left to right blood flow through ductus arteriosus, followed by functional and anatomical closure of ductus arteriosus [1].

Its incidence is inversely related to the gestational age. Ductus arteriosus remains patent at 4 days of age in 10% of early and late preterm neonates and up to 80–90% in extremely premature neonates [2]. Spontaneous closure might occur in a high proportion of preterm neonates [3], however in cases of failure of closure, persistent PDA in preterm infants can lead to higher morbidity and cause complications including respiratory failure, lower survival rate, increased risk of intracranial hemorrhage, chronic lung disease and necrotizing enterocolitis, and it is one of the main factors ameliorating the mortality of premature infants [4].

In order for a PDA to be symptomatic, it needs to be hemodynamically significant, which can be determined by M-Mode Echocardiography showing a ratio of left atrium diameter (LA) to aortic root diameter (Ao), which if more than 1.4, signifies a Hemodynamically significant PDA (HsPDA). Moreover, evidence of substantial shunt suggested by a reversal of blood flow in diastole in abdominal aorta also signifies HsPDA [3].

Comparing both drugs pharmacologically; paracetamol acts on the peroxidase segment of the prostaglandin synthetase enzyme and inhibits its activity, whereas ibuprofen acts by nonselective inhibition of cyclooxygenase enzymes, which marks their pharmacologic basis of closure of PDA. The peroxidase requires a 10-fold lower concentration to be activated compared to cyclooxygenase, hence hypothetically more effective in cases associated with neonatal hypoxia [5, 6].

Based on the decision to treat the condition, it has been a topic of debate among the neonatologists. Various researches have been carried out around the globe to find a better treatment option among paracetamol, ibuprofen and indomethacin [7], although a universal consensus over the management of PDA could not be found, hence practices over the management vary among the healthcare facilities ranging from prophylactic treatment to selective surgical treatment of the condition [8].

Considering the lack of studies done on this topic in healthcare settings within the Khyber Pakhtunkhwa Province of Pakistan, this study aims to find out and compare the effectiveness of paracetamol and ibuprofen in management of PDA in our healthcare setting to conclude a better management option for the condition.

## Objective

To find and compare the effectiveness of paracetamol and ibuprofen in the closure of patent ductus arteriosus in preterm neonates.

## Materials and methods

This randomized controlled trial was conducted in the Department of Nursery (Special Care Baby Unit) and Neonatal Intensive Care Unit (NICU) at Khyber Teaching Hospital, Peshawar, Pakistan from 10th April 2024 to 10th October 2024, after obtaining approval from the concerned authorities, Institutional Ethical Review Board (797/DME/KMC), and NIH Trial Registry (NCT06601114) https://clinicaltrials.gov/study/NCT06601114. After obtaining an informed consent from parents, a total of 300 neonates of both genders with patent ductus arteriosus (PDA) confirmed through echocardiography were enrolled after meeting the inclusion criteria. Neonates were eligible for the study if they had a gestational age between 30 and 37 weeks, a birth weight of ≥ 1250 g, and a postnatal age of 48 to 96 h. Inclusion also required echocardiographic evidence of patent ductus arteriosus (PDA), defined by at least one of the following: a duct size > 2 mm, a left atrium-to-aorta ratio > 1.4, end-diastolic reversal of blood flow in the aorta, or poor cardiac function. These echocardiographic findings were accompanied by clinical signs consistent with PDA.

**Fig. 1:**
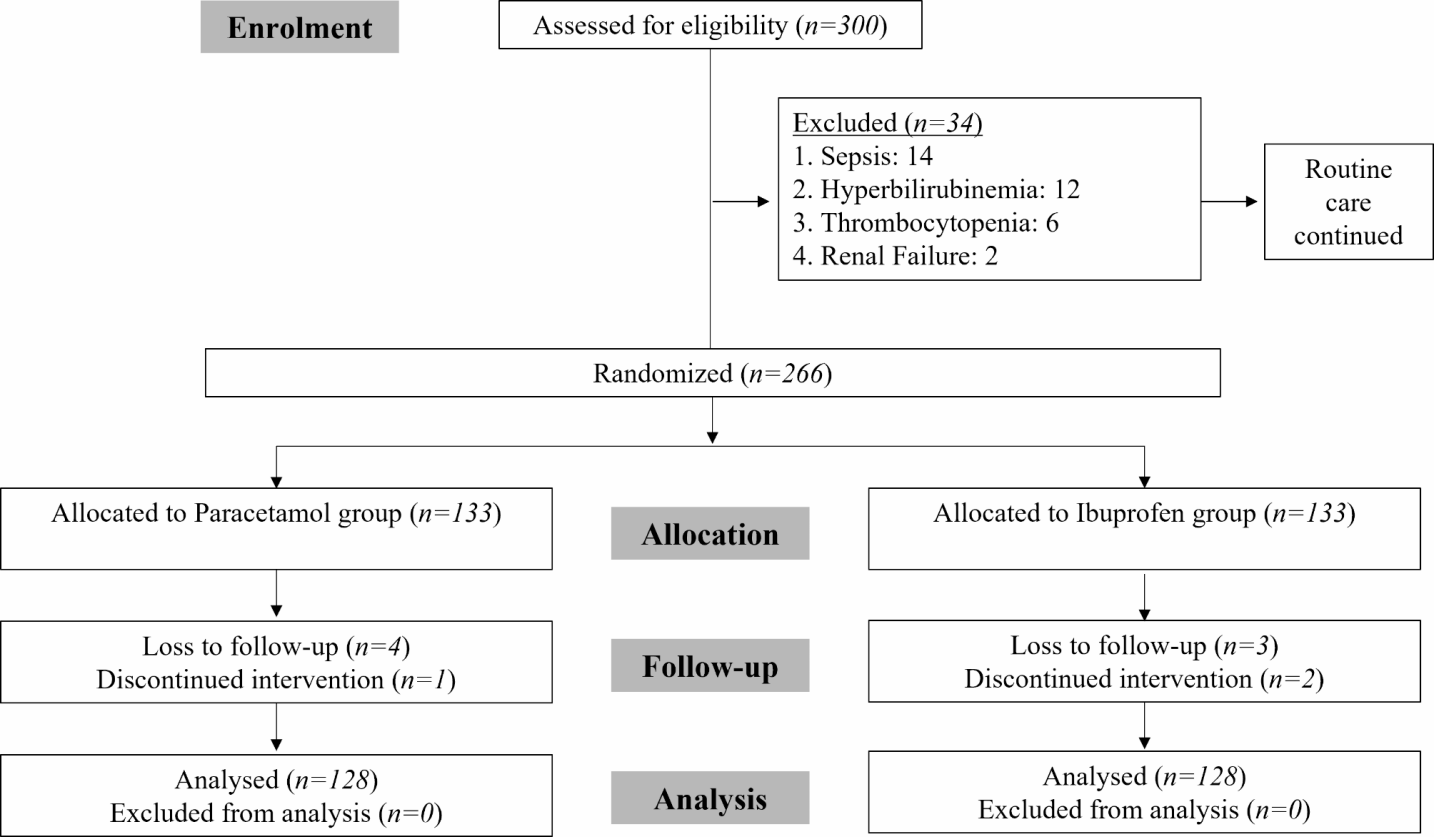
Study Flow as per CONSORT recommendation

Neonates with contraindications to paracetamol or ibuprofen, such as pre-existing liver or renal impairment, severe sepsis, necrotizing enterocolitis, major congenital anomalies, or those already receiving treatment for PDA or with incomplete medical records, were excluded. The sample size was calculated using the WHO sample size calculator with a 95% confidence level, absolute precision of 10%, and proportions of 82.1% and 75.8% for the paracetamol and ibuprofen groups, respectively, derived from a previous study [9], resulting in a needed sample size of 254. Recruitment occurred between 10th April 2024 and 1st September 2024, using non-probability consecutive sampling with a 1:1 allocation ratio (Fig. 1). Block randomization was performed, with block size of 4, and allocation was concealed using sealed envelopes to ensure blinding. The randomization sequence was generated by the study coordinator, while recruitment and enrollment were conducted by the clinical team, and a separate team allocated intervention. Blinding was implemented for outcome assessors and data analysts, although healthcare providers administering treatments were not blinded due to protocol differences. Group A received oral paracetamol (15 mg/kg every 6 h for 3 days), and Group B received oral ibuprofen (10 mg/kg initially, followed by 5 mg/kg at 24 and 48 h). Routine management, including broad spectrum antibiotics such as ampicillin and gentamicin (as first line antibiotics therapy), and intravenous fluids, was provided according to departmental policies. Effectiveness was defined as the complete closure of PDA confirmed via echocardiography at the end of the treatment. Data were collected on a structured proforma and analyzed using SPSS version 22. Frequencies and percentages were calculated for qualitative variables such as gender and efficacy, while means ± SD were presented for quantitative variables like age, weight, and gestational age. The Chi-Square test was applied to compare effectiveness between groups, taking *p* ≤ 0.05 as significant. Stratification by age, gender, gestational age, and weight was performed, followed by post-stratification Chi-Square analysis, with *p* ≤ 0.05 considered statistically significant.

## Results

This study compared Groups A and B, each with 128 patients, focusing on demographics, gender (Table 1), and efficacy (Table 2). Group A had a slightly younger average age (71.79 ± 13.10 h) and higher weight (1638.58 ± 140.60 kg) compared to Group B (Table 1). Gender distribution was similar in both groups, with a higher proportion of females (66.4% in Group A and 62.5% in Group B, Table 1).

When examining efficacy, Group A had significantly better outcomes (83.6%) than Group B (70.3%) with a P value of 0.011 **(**Table 2). Stratification by gender revealed that males in Group A had a notably higher efficacy rate (88.4%) compared to Group B (62.5%), while the difference for females was less pronounced (81% in Group A vs. 75% in Group B), with no significant P value (*P* = 0.357) (Table 3).

Further stratification by gestational age and weight showed trends supporting Group A’s higher efficacy. Neonates born at 30–35 weeks had significantly better outcomes in Group A (84.5% vs. 71.1%, *P* = 0.022). For neonates weighing more than 1500 g, Group A also showed superior efficacy (83.5% vs. 65.5%, *P* = 0.005), emphasizing the effectiveness of interventions in Group A, particularly for heavier and more mature infants (Table 3).

**Table 1:** Demographics

Category	Subcategory	Group A (*n* = 128)	Group B (*n* = 128)	*P* Value
Demographics	Age (hours)	71.79 ± 13.10	73.40 ± 11.81	-
	Weight (g)	1638.58 ± 140.60	1612.20 ± 141.80	-
	Gestational age at birth (weeks)	33.31 ± 1.99	33.51 ± 2.10	-
Gender Distribution	Male	43 (33.6%)	48 (37.5%)	-
	Female	85 (66.4%)	80 (62.5%)	-
	Total	128 (100%)	128 (100%)	-

**Table 2:** Efficacy

Category	Subcategory	Group A (*n* = 128)	Group B (*n* = 128)	*P* Value
Efficacy Comparison	Yes	107 (83.6%)	90 (70.3%)	0.011
	No	21 (16.4%)	38 (29.7%)	
	Total	128 (100%)	128 (100%)	

**Table 3:** Stratification analysis

Category	Subcategory	Group A (*n* = 128)	Group B (*n* = 128)	*P* Value
Efficacy Stratification by Age	48–72 h: Yes	60 (87%)	46 (74.2%)	0.063
	48–72 h: No	9 (13%)	16 (25.8%)	
	> 72 h: Yes	47 (79.7%)	44 (66.7%)	0.103
	> 72 h: No	12 (20.3%)	22 (33.3%)	
Efficacy Stratification by Gender	Male: Yes	38 (88.4%)	30 (62.5%)	0.004
	Male: No	5 (11.6%)	18 (37.5%)	
	Female: Yes	69 (81%)	60 (75%)	0.357
	Female: No	16 (19%)	20 (25%)	
Efficacy Stratification by Gestational Age	30–35 weeks: Yes	87 (84.5%)	64 (71.1%)	0.022
	30–35 weeks: No	16 (15.5%)	26 (28.9%)	
	> 35 weeks: Yes	20 (80.0%)	26 (68.4%)	0.356
	> 35 weeks: No	5 (20.0%)	12 (31.6%)	
Efficacy Stratification by Weight	≤ 1500 g: Yes	26 (83.9%)	31 (81.6%)	0.803
	≤ 1500 g: No	5 (16.1%)	7 (18.4%)	
	> 1500 g: Yes	81 (83.5%)	59 (65.5%)	0.005
	> 1500 g: No	16 (16.5%)	31 (34.4%)	

## Discussion

In this study, we compared the effectiveness of paracetamol and ibuprofen in closing the patent ductus arteriosus (PDA) in preterm neonates. The study included neonates aged 48 to 96 h, with a mean age of approximately 72 h for both groups. The mean birth weight was around 1638 g in Group A (paracetamol) and 1612 g in Group B (ibuprofen), and the mean gestational age at birth was roughly 33 weeks for both groups. Notably, female neonates were more prevalent in both groups.

The results demonstrated a higher efficacy of paracetamol compared to ibuprofen in the closure of PDA. Specifically, 83.6% of neonates in Group A achieved PDA closure, compared to 70.3% in Group B, with a statistically significant difference (*P* = 0.011). This indicates that paracetamol is more effective than ibuprofen for this condition in comparatively mature preterm neonates.

Several factors could explain these findings. First, the pharmacodynamics and pharmacokinetics of paracetamol may make it more effective in the neonatal population. Paracetamol may have a more favorable safety profile and fewer adverse effects on the gastrointestinal tract, which can be particularly sensitive in preterm infants. Additionally, paracetamol’s mechanism of action might be more suitable for achieving PDA closure in neonates, possibly due to its effect on prostaglandin synthesis pathways.

The dominance of female gender in both groups aligns with existing literature suggesting gender differences in the prevalence and outcomes of PDA, although the underlying reasons for this are not entirely clear. Hormonal and genetic factors may play a role, and further research is needed to elucidate these mechanisms.

A promising area of investigation is the combined use of paracetamol and ibuprofen for PDA closure. Although both drugs inhibit prostaglandin synthesis, they act through different mechanisms and target different pathways. Emerging evidence suggests that combination therapy may have a synergistic effect, offering higher closure rates compared to monotherapy [10]. Initial clinical data indicate that combination therapy could be more effective, particularly for hemodynamically significant PDA, but further studies are needed to confirm its safety and efficacy [11].

The significant difference in efficacy between the two drugs suggests that paracetamol could be considered a preferable first-line treatment for PDA in preterm neonates, potentially leading to better clinical outcomes and reduced need for surgical intervention. These findings contribute to the ongoing efforts to optimize the management of PDA in this vulnerable population, highlighting the importance of selecting the most effective and safe therapeutic options.

According to Lago et al. [12], ibuprofen demonstrates comparable efficacy and safety to indomethacin in closing patent ductus arteriosus (PDA) in preterm infants with respiratory distress syndrome. The study highlights that ibuprofen has a lesser impact on renal function, specifically in terms of urine output and fluid retention, making it a potentially favorable choice in this clinical context.

Klerk et al. [13] report that the spontaneous closure rates of the ductus arteriosus in preterm infants vary significantly based on patient characteristics, with lower gestational ages and birth weights correlating with lower closure rates. The study emphasizes the importance of accurately predicting spontaneous closure to determine the optimal management strategy for PDA in preterm neonates. Additionally, it highlights the possibility of the ductus arteriosus reopening post-closure due to factors like infection or inflammation, underscoring the need for ongoing monitoring and care in these vulnerable populations.

In a related study by Bagheri et al. [9] found no significant difference in the effectiveness of oral acetaminophen versus oral ibuprofen in closing PDA in preterm neonates. This randomized clinical trial involving 120 infants, concluded that both medications showed similar efficacy in treating PDA in preterm infants with a gestational age of less than 37 weeks. Building on this, Allegaert et al. [14] propose paracetamol as a viable alternative to non-selective cyclo-oxygenase inhibitors for inducing closure of the PDA in preterm neonates, due to the limitations and risks associated with these traditional medications.

Hirt et al. [15] provide further insights through a population pharmacokinetic analysis of ibuprofen in preterm neonates. Their study found that ibuprofen clearance increased significantly with postnatal age, but not with gestational age. A correlation was established between the area under the curve (AUC) of ibuprofen and the closure rate of PDA, suggesting a threshold AUC for optimal effectiveness. Additionally, the researchers also proposed dosing regimens based on postnatal age to achieve the desired AUC, thereby enhancing treatment efficacy.

Yaman’s study [16] contributes additional evidence by demonstrating that both ibuprofen and paracetamol are effective in treating hemodynamically significant PDA in term infants, with no statistically significant difference in success rates. These findings provide valuable insights into the potential efficacy of paracetamol and ibuprofen in closing PDA, supporting their use as alternatives to indomethacin and ibuprofen in preterm infants.

Thomas et al. [17] adds to the discussion by comparing ibuprofen and indomethacin, both of which exhibit comparable efficacy in PDA closure. However, ibuprofen demonstrates additional advantages, including lower serum creatinine levels, increased urine output, and fewer adverse effects on organ blood flow and vasoconstriction. This positions ibuprofen as a potentially safer alternative in clinical practice.

Further supporting the use of paracetamol, Bardanzellu et al. [18] present evidence showing that paracetamol offers PDA closure rates comparable to those of traditional NSAIDs, but with reduced toxicity. These findings align with the growing trend toward safer pharmacologic options for PDA management.

Finally, Yücel and Şahin [19] affirm that medical closure of PDA in preterm neonates can be achieved using indomethacin, ibuprofen, or paracetamol. Their findings support the use of paracetamol and ibuprofen as effective alternatives to indomethacin, further reinforcing the expanding range of pharmacologic options available for managing PDA in preterm neonates.

## Conclusion

Based on the results of our study, we conclude that paracetamol is more effective than ibuprofen in closing patent ductus arteriosus (PDA) in preterm neonates. The higher efficacy rate observed with paracetamol suggests that it may be a preferable first-line treatment for PDA in this population. Additionally, the comparable safety profiles of both medications, along with the observed female gender dominance in both groups, support the use of paracetamol as a viable alternative to ibuprofen for managing PDA in preterm neonates. Further research is warranted to explore the underlying mechanisms and to confirm these findings in larger, more diverse cohorts.

## Data Availability

No datasets were generated or analysed during the current study.
